# Pilot Findings Indicate a Cognitive Behavioral Healthy Lifestyle Intervention for PTSD Improves Sleep and Physical Activity

**DOI:** 10.3390/brainsci13111565

**Published:** 2023-11-08

**Authors:** Jeffrey Kibler, Mindy Ma, Jacquelyn Hrzich, Jessica Choe

**Affiliations:** 1 Department of Clinical and School Psychology, College of Psychology, Nova Southeastern University, 3300 S. University Dr., Ft. Lauderdale, FL 33328, USA; mmindy@nova.edu; 2 Psychological Dimensions, 6595 S. Dayton St., Greenwood Village, CO 80111, USA; jh2573@mynsu.nova.edu; 3 James A. Haley Veterans’ Hospital, 13000 Bruce B. Downs Boulevard, Tampa, FL 33612, USA; jc3882@mynsu.nova.edu

**Keywords:** PTSD, health behaviors, healthy lifestyle, intervention, physical activity, sleep

## Abstract

Research has indicated strong associations between post-traumatic stress and cardiovascular disease (CVD) risk. Individuals with post-traumatic stress disorder (PTSD) tend to show patterns of elevated CVD risk earlier in life than the general population. The need for developing effective interventions for CVD risk reduction in PTSD is increasingly evident. The purpose of the present pilot study was to examine the effects of a healthy lifestyle intervention that addresses CVD-related heath behaviors (physical activity, sleep, stress) among civilian adults with PTSD. Participants were randomized to the healthy lifestyle intervention condition or a wait-list control. A total of 22 women completed the protocol (11 per group). The mean age was 32 (SD ± 14). Evaluations were conducted before and after the 12-week intervention program in the experimental group, and 12 weeks apart for the control group, and included standardized self-report measures of sleep, physical activity, and general stress. The healthy lifestyle group showed an increase in the amount of sleep pre to post (mean of 1.2 h per night), which was significantly different to the control group, who had no change (*p* < 0.05; effect size = 1.41). Notable pre to post increases in physical activity were observed between the intervention group (mean increase = 115.8 min over 7 days) and control condition (mean = 4.5 min over 7 days); however, this effect was not significant in the small sample (effect size = 0.70). These preliminary findings suggest that a healthy lifestyle intervention is feasible and can produce desired changes in target behaviors/outcomes.

## 1. Introduction

A growing body of research has indicated that post-traumatic stress disorder (PTSD) is associated with disruptions in health behaviors that may, in turn, increase cardiovascular disease (CVD) risk [1,2,3,4]. Numerous studies indicating those with PTSD are at greater CVD risk, earlier in life e.g., [2,5,6,7,8]. Compared to the cumulative evidence concerning elevated CVD risk in PTSD, relatively little research has addressed CVD risk reduction in this population [3,9,10,11]. One study revealed that veterans whose PTSD symptoms improved while treated in VA PTSD specialty clinics did not show significant improvement in CVD risks [3]. This suggests that adjunctive treatments, such as health behavior interventions, may be necessary as supplements to traditional psychotherapy for PTSD in order to reduce CVD risks. These types of health behavior interventions would need to specifically address the physical health and physiological aspects of PTSD symptomatology that may not be adequately targeted with standard mental health treatments [3,9,11].

Several unique aspects of PTSD symptom presentation may account for the elevated CVD risks that have been observed in this population. For example, heightened awareness or fear of bodily arousal symptoms, such as increased heart rate or shortness of breath, have been associated with less physical activity and lower ratings of exercise motivation in PTSD [12,13]. When these bodily responses occur in the context of anxiety reactions, the fear of bodily arousal can be conceptualized as anxiety sensitivity, and high anxiety sensitivity is commonly reported among those with PTSD [14,15]. Anxiety sensitivity may lead to an avoidance of exercise. Conversely, there is experimental evidence that engaging in physical activity can decrease anxiety sensitivity [12,16], thereby reversing the negative cycle of avoidance at least temporarily. These effects are theorized to occur through exposure and desensitization to internal arousal cues [16]. Potential fear of arousal symptoms may underscore the need to begin slowly when implementing physical activity for individuals with PTSD who have been sedentary. Research and health behavior theories support the notion that abrupt increases in activity are not as likely to be maintained [17]. Such abrupt changes may be more difficult when one has hypersensitivity to arousal [17]. Therefore, we have designed our experimental intervention to be sensitive to the specific needs of participants with PTSD. Hall et al. [18] indicated that increasing physical activity is feasible, and can enhance aerobic performance and metabolic measures among older military veterans with PTSD. Our present approach permits a preliminary examination of a structured cognitive behavioral approach to physical activity and other health-related behaviors among civilians with PTSD.

Recurring trauma-related nightmares in PTSD and consequent behavioral conditioning of sleep avoidance are disruptive, prolonging sleep latency and contributing to irregular bedtimes [19,20,21]. Sherrer et al. [3] demonstrated that sleep disorders mediated PTSD/CVD associations. Thus, sleep interventions may enhance CVD risk-reduction efforts [22,23]. Psychotherapies for PTSD have not typically addressed sleep problems directly or systematically, and sleep outcomes following therapy have not generally been assessed [24,25]. The limited number of CBT interventions that have focused specifically on sleep in PTSD have been encouraging [26,27]. Therefore, additional research on this area of treatment may further inform the potential for health behavior intervention to impact health in PTSD. Relationships of sleep with physical activity and stress e.g., refs. [28,29] may help in explaining the potential role of sleep in associations between PTSD and CVD risk [3].

Stress-related and emotional factors have been identified as early candidate mechanisms for PTSD/CVD relationships, with various responses to acute and chronic stress being implicated in this relationship [4,30]. For example, PTSD severity was shown to be related to delayed cardiovascular recovery [31]. Delayed cardiovascular recovery in the lab has been associated with greater carotid intima–media thickness, as well as elevated resting blood pressure measured 3 years after lab assessment of recovery [32,33]. Perceived threat (a pervasive maladaptive cognitive response in PTSD) mediated these physiological disruptions [31]. Research has identified cognitive appraisals, including perceived threat, as key factors to address in PTSD [34]. Although the implications of cognitive appraisals for health behaviors in PTSD have not been adequately researched, the implications for stress are evident, and Lazarus and colleagues’ Cognitive Appraisal Theory suggests that the types of disrupted cognitions evident in PTSD may interfere with the pursuit of healthy behaviors such as physical activity [35]. Interventions to address cognitive appraisals may reduce stress-related CVD risk in PTSD by enhancing the ability to cognitively cope with stressors and engage in healthy behaviors.

The purpose of the present paper is to present preliminary pilot data on the effects of a cognitive behavioral healthy lifestyle intervention among civilian adults with PTSD. Participants who were randomized to either the healthy lifestyle intervention condition or a wait-list control group were evaluated for changes in physical activity, sleep, and stress. It was hypothesized that the healthy lifestyle intervention would result in greater improvements in physical activity, sleep, and stress.

## 2. Methods

### 2.1. Participants

A total of 22 participants completed the study (11 per condition). All participants in this sample were women, although the study was also open to men. The mean age was 32.0 years (SD = 14.0). The breakdown of race/ethnicity was as follows: 10 Caucasian, non-Hispanic; 3 African American; 3 Haitian; 2 Hispanic; 2 Caribbean Black; and 2 Asian American. Table 1 depicts the demographic data by participant group. Adults aged 18 and older were recruited from the community, local clinics, and via online advertisements. Criteria for inclusion were no history of major chronic illness, PTSD related to a trauma event occurring at least one year prior to the study, the presence of at least one of three health risks (physical inactivity, overweight, or sleep disruption), and ability to exercise at a moderate level. Concurrent psychotherapy was anticipated for participants and was, therefore, not exclusionary. Comorbid psychiatric conditions (e.g., depression, personality disorders, substance use disorders) were not exclusionary, as this would result in an unnatural narrow selection of participants with PTSD. Participants who qualified for the study were randomized to the healthy lifestyle intervention condition or a wait-list control condition. Outcome evaluations were conducted before and after the 12-week intervention program in the experimental group, and 12 weeks apart for the control group. Written informed consent was obtained from all participants. This study was approved by the appropriate university IRB, and conformed to the principles embodied in the Declaration of Helsinki.

### 2.2. Measures

#### 2.2.1. Pittsburgh Sleep Quality Index (PSQI)

The PSQI is a self-report survey with 19 items [36] that was utilized to assess sleep quality and quantity, and sleep efficiency, in the past 4 weeks. Responses are used to determine a global score that ranges from 0 to 21, with higher values representing worse sleep quality (5 or greater represents impaired sleep quality). Studies have indicated high sensitivity and specificity for the PSQI in predicting insomnia, high internal consistency, and high test–retest reliability and convergent validity with other sleep measures [36,37].

#### 2.2.2. The 7-Day Physical Activity Recall

This physical activity measure was originally developed for use in the Stanford Heart Prevention Program. It includes an assessment of the amount of time spent in moderate and intense aerobic exercise in the past week (physical activity outcome variable).

#### 2.2.3. Weekly Stress Inventory Short Form (WSI)

The WSI was administered to measure self-reported stress. The WSI is a 25-item list of events that are usually perceived as stressful [38]. Participants rate the amount of stress caused by the event, rating items on an 8-point Likert scale, with values ranging from 0 (did not occur) to 7 (extremely stressful). The WSI has been found to have adequate internal consistency, reliability, and validity [38].

#### 2.2.4. The Post-Traumatic Stress Checklist 5th Edition (PCL-5)

To verify the participants’ PTSD symptoms, the PCL-5 was administered at baseline. The PCL-5 is a 20-item self-report measure that assesses the 20 DSM-5 symptoms of PTSD [39]. Items are rated on a 5-point scale ranging from 0 (not at all) to 4 (extremely) over the past 30 days. Good internal reliability, convergent validity with the CAPS, and diagnostic efficiency of the PCL have been demonstrated across various trauma populations [39].

## 3. Procedures

Participants were recruited from university- and community-based clinics, private practices, and from the community using written and online notices/flyers. Potential participants called a private central phone number to express interest in the study. Volunteers were then screened by phone to assess basic study inclusion and exclusion criteria, and to provide additional details about the study. Randomization to study conditions was performed by the project coordinator, using the flip of a coin, following the initial phone contact. At the baseline/pre-intervention assessment, informed consent and additional screening assessments were administered to further evaluate and verify eligibility based on PTSD symptoms and the presence of at least one risk factor targeted by the intervention (e.g., physical inactivity, overweight status, sleep difficulties). Data collection for the study outcomes was conducted at pre- and post-intervention sessions. Participant compensation consisted of USD 50 Walmart gift cards for each of the pre- and post-assessment sessions, and USD 5 in gift cards for each intervention session.

The interventionists for the healthy lifestyle program were advanced doctoral students with experience delivering CBT-based health interventions. The interventionists were familiar with the lifestyle intervention literature, and developed a thorough understanding of the protocol for PTSD patients by reviewing the manual and meeting with the PI for training.

### Healthy Lifestyle Intervention

The healthy lifestyle intervention was a 12-session program with sequential but overlapping modules for increasing physical activity, improving sleep, and improving stress management. The sessions were conducted individually, and were held weekly for 90 min. The first module included psychoeducation on physical activity (health benefits of physical activity and the health risks associated with physical inactivity and overweight), as well as the implementation of an exercise regimen and some of the inherent challenges. Participants were encouraged to begin a physical activity regimen consistent with recommendations for a healthy lifestyle (aerobic exercise at least 20 min 2–3 times per week). However, the interventionists instructed participants to work toward exercise goals that could be incorporated into their lifestyles and maintained. In addition, participants walked for 20 min with the interventionist during intervention sessions 2–5. 

Sleep-focused intervention began in session 5, and continued into session 8. In the fifth session, an overview of common sleep problems was introduced, and the participant addressed any sleep difficulties they had experienced. A rationale for the treatment used in our protocol was provided. During sessions 6 through 8, cognitive behavioral strategies for assisting with sleep improvement were incorporated and discussed. In session 6, participants also identified a problematic nightmare (where applicable) and initiated the process of imagery rehearsal therapy by beginning to develop and write a modified version of the dream. In addition to a discussion of insomnia treatment strategies in sessions 7 and 8, participants continued to develop their modified dream and rehearse it in the sessions. Participants were instructed to rehearse the modified dream daily for 5–20 min.

Sessions 9–12 focused on stress management, relapse prevention, and other issues related to termination of the program. During session 9, the interventionist provided an overview of the cognitive behavioral model of stress and initiated a discussion of various aspects of the model (e.g., negative thoughts, negative emotions, and their relationship to behavior). Between sessions 9 and 10, the participants were asked to self-monitor negative thoughts to provide a basis for follow-up discussion in session 10. Session 10 then focused on common patterns of negative thoughts and associated negative feelings. During session 10, participants were familiarized with thought logs [40], which use personal situations identified by participants to assist in identifying relationships between negative thoughts, negative feelings, and behavioral outcomes, and in constructing responses to stressful situations that are more adaptive. Participants were given the homework to complete two or more thought logs between sessions 10 and 11, and the results were discussed during session 11. During session 12, discussion of thought logs and stress coping was continued for the first part of the session. A portion of the final session included a review/overview of the skills learned, discussion of risks for relapse and skills for relapse prevention, and discussion of any concerns associated with termination of the intervention program.

## 4. Results

### 4.1. Descriptive Data

The mean level of PTSD symptoms on the PCL at baseline was 44.5 (SD = 15.8), which greatly exceeds the recommended cut-point of 33 for probable PTSD. The PTSD symptom levels did not differ by study condition/group. Trauma types/incidents identified as primary index traumas included sexual assault or abuse (six participants); physical assault or abuse (three); automobile accident (three); witnessing violence (three); other accident, injury, or life-threatening illness (three); sudden unexpected death of a loved one (two); combat (one); and direct experience of the 911 tragedy (one). Preliminary analyses to compare the study groups on demographic variables revealed that there were no significant group differences in age, race/ethnicity, education, or income. A *t*-test was used for comparison by age, and chi-square tests of independence were utilized to compare groups on the other demographic parameters. These data and comparisons by study group are illustrated in Table 1.

### 4.2. Group Differences in Primary Outcome Measures

Primary analyses involving standard between-group comparisons (one-way ANOVA with participant group as the single independent variable) revealed that the healthy lifestyle group showed an increase in the amount of sleep from pre- to post-assessment (mean of 1.2 h per night), which was significantly different to the control group, who had no change (*F* = 5.12; *p* < 0.05; effect size = 1.41). The results for the sleep analyses are depicted in Table 2. 

**Figure 1:**
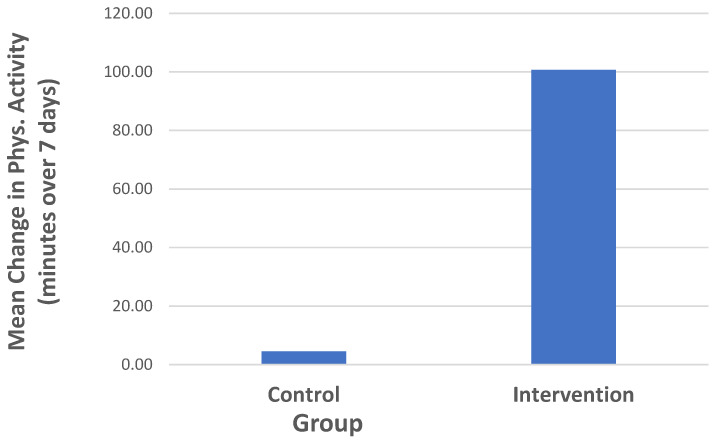
Change in physical activity (pre to post) by group.

Notable differences in the pre to post changes in physical activity were observed between the intervention group (mean increase = 115.8 min over 7 days) and control condition (mean = 4.5 min over 7 days). However, this effect was not significant in the small sample (*F* = 2.05; *p* = 0.17; effect size = 0.70). The results from the physical activity analyses are depicted in Table 3, and are illustrated in Figure 1. Small improvements in stress were detected in the intervention group (mean change of 3.3 points in the WSI), with less improvement in the control group (mean change of 1.7). However, this difference was not significant (*F* = 0.18, *p* = 0.89).

## 5. Discussion

The present results provide preliminary support for the feasibility and positive health effects of a relatively short-term healthy lifestyle intervention among women with PTSD, CVD risk, and no diagnosed medical illnesses. The intervention program had the greatest impact on the amount of sleep and physical activity reported. Given the prior data on sleep disruption and lower physical activity in PTSD [13,19], these outcomes are interpreted as major changes that would likely enhance health and quality of life if the changes are maintained by individuals with PTSD.

The increased amount of sleep in the intervention group (mean of 72 min per night) was considerable, and exceeds the improvements in sleep amount of approx. 30–35 min per night observed in other intervention studies for PTSD [41,42]. One potential explanation for this positive finding is that addressing sleep in conjunction with other health behaviors may confer a synergistic effect that is greater than the impact of each component alone [43,44]. One study showed a positive association between exercise intervention and improved sleep in PTSD [45]. The interaction of physical activity with sleep may have affected the results of the present study, as the physical activity intervention took place in the first phase of the program and may have served as a catalyst for better improvements in sleep. The large effect for physical activity, while not statistically significant in the small sample, exceeded our expectations for improvements in this population during the 12-week period. It is advisable to increase physical activity gradually when intervening [17], and this was incorporated into our approach. Nonetheless, we found that participants gravitated toward greater change, increasing activity faster than we anticipated. Our findings are encouraging with regard to the potential for engaging patients with PTSD in physical activity. However, caution is advised in terms of expecting these types of large changes consistently; additional research is needed to evaluate whether the changes we observed are maintained with a larger sample and whether the changes can be sustained beyond the 12-week intervention period.

The data on women for the present study may provide perspectives on any unique factors related to physical activity implementation for women with PTSD [46]. One hypothesis concerning weight regulation in PTSD is that, among abuse survivors, higher weight can serve as a protective mechanism [47]. Women are more likely to be victims of physical and sexual abuse, and may feel more empowered to ward off abuse if their weight makes them more physically daunting to a perpetrator [47]. Although we did not have data pertaining to these phenomena in the present study, we were mindful of these potential barriers to physical activity and maintained an open and accepting approach when discussing participants’ perspectives on exercising. We are confident that our sensitivity to these issues enhanced our ability to engage participants. In all, our preliminary findings address the need for data on health behavior intervention in women with PTSD and are encouraging; the results suggest that women with PTSD have a high capacity for enhancing physical activity. It is notable, however, that our study did not include data from men. This must be accounted for when interpreting the implications of our findings. While the literature generally supports the notion that women and men with PTSD do not differ in terms of CVD risk [48,49], until there are data available from both women and men, we cannot evaluate whether there could be differential effects of the present intervention by gender. The points we raised about weight regulation for victims of PTSD may or may not apply equally to physical activity for women and men [46]. Obtaining a balanced sample by gender would also help us to explore these mechanistic issues.

Additional data are needed to evaluate whether a health behavior program will produce consistent improvements in stress that exceed standard care. We observed small improvements in stress overall, and additional data from a larger sample are needed to determine whether this will be a consistent finding. One consideration in terms of interpreting our findings is that stress management was the last module in our health behavior program. It is possible that participants had more time to incorporate skills related to physical activity and sleep into their lifestyles, whereas the stress management module occurred within the weeks preceding the post assessment. Incorporating follow-up periods of several months in future studies would permit better detection of longer-term changes in stress and other outcomes. Preliminary findings from other studies regarding stress reduction are encouraging, in that CBT-based approaches may lead to improvements in stress levels and PTSD symptoms [50,51].

Aside from the inherent primary limitation of the low sample size in this small pilot study, another limitation is the self-report nature of the outcome variables. Further research incorporating objective sleep and physical activity monitoring e.g., ref. [28] will provide a stronger basis for evaluating the changes identified in the present study. In addition, physiological indices of stress would provide a good complement to subjective stress ratings, and may confer additional perspectives on the experience and significance of stress and stress reduction. Additional markers of CVD risk will also help in expanding this area of research by evaluating the potential to reduce CVD risk in PTSD. Given our focus on CVD-related health behaviors, we did not include a full host of traditional CVD risk variables (e.g., lipids, blood pressure, body mass). Inclusion of a more in-depth analysis of CVD risk, potentially including innovative measures of cardiovascular function, would enhance the ability to evaluate positive changes in health risk resulting from interventions such as ours.

## 6. Conclusions

Despite the limitations, the present study represents a solid preliminary step in developing and testing a unique approach to intervention in the cardiovascular health consequences of PTSD. Our approach addresses the need for interventions that specifically target the unique physical health and physiological aspects of PTSD symptomatology, which may not be adequately targeted with standard mental health treatments [3,10,11]. Further testing of this paradigm will likely build on our findings and further evaluate the potential for adjunctive treatments, such as health behavior interventions, to supplement traditional psychotherapy for PTSD in order to reduce CVD risks.

## Figures and Tables

**Table 1:** Demographic data by participant group (experimental intervention vs. control group).

	Participant Group		Test of Significance
Variable	Health Behavior Intervention	Control Group	*p* Value
Mean Age (SD)	32.8 (13.6)	31.8 (15.1)	0.87
Race/ethnicity (# per group):			0.46
Hispanic White	1	1	
Non-Hispanic White	5	5	
Carribean Black (not Hispanic)	0	2	
African American	1	2	
Asian	2	0	
Haitian	2	1	
Education (# per group):			0.32
High school or equivalent	3	3	
Associate’s Degree	1	0	
Bachelor’s Degree	4	2	
Master’s Degree	3	3	
Other	0	3	
Marital Status (# per group)			0.38
Single	6	8	
Married	1	1	
Divorced, separated, or widowed	4	2	
Income (# per group)			0.74
<USD 5000	1	3	
USD 5000–USD 10,000	1	1	
USD 10,001–USD 15,000	1	0	
USD 15,001–USD 20,000	1	1	
USD 20,001–USD 30,000	0	0	
USD 30,001–USD 40,000	3	1	
USD 40,001–USD 50,000	0	1	
USD 50,001–USD 75,000	1	1	
USD 75,001–USD 100,000	1	2	
USD 100,000+	0	1	

**Table 2:** Results of the ANOVA test for change in sleep amount from pre to post intervention by study group.

Analysis of Variance					
Source	Sum of Squares	df	Mean Square	F	Sig.
Between groups	7.860	1	7.860	5.118	0.036
Within groups	29.181	20	1.536		
Total	37.042	21			

**Table 3:** Results of the ANOVA test for change in physical activity from pre to post intervention (minutes over 7 days) by study group.

Analysis of Variance					
Source	Sum of Squares	df	Mean Square	F	Sig.
Between groups	64,776.648	1	64,776.648	2.048	0.169
Within groups	600,898.352	20	31,626.229		
Total	665,675.000	21			

## Data Availability

The data are not available for sharing at this time, as these are pilot data and the project will be ongoing for several years. Data will be shared following project completion, per the plan outlined to the funder (NIH).
